# Liposomes-In-Hydrogel Delivery System Enhances the Potential of Resveratrol in Combating Vaginal Chlamydia Infection

**DOI:** 10.3390/pharmaceutics12121203

**Published:** 2020-12-11

**Authors:** May Wenche Jøraholmen, Mona Johannessen, Kirsten Gravningen, Mirja Puolakkainen, Ganesh Acharya, Purusotam Basnet, Nataša Škalko-Basnet

**Affiliations:** 1 Drug Transport and Delivery Research Group, Department of Pharmacy, Faculty of Health Sciences, UiT The Arctic University of Norway, Universitetsveien 57, 9037 Tromsø, Norway; natasa.skalko-basnet@uit.no; 2 Research Group for Host Microbe Interactions, Department of Medical Biology, Faculty of Health Sciences, UiT The Arctic University of Norway, Universitetsveien 57, 9037 Tromsø, Norway; mona.johannessen@uit.no; 3 Department of Microbiology & Infection Control, University Hospital of Northern Norway, Sykehusveien 38, 9019 Tromsø, Norway; KirstenMidttun.Gravningen@fhi.no; 4 Department of Virology, University of Helsinki and Helsinki University Hospital, 00014 Helsinki, Finland; mirja.puolakkainen@helsinki.fi; 5 Department of Obstetrics and Gynecology, University Hospital of North Norway, Sykehusveien 38, 9019 Tromsø, Norway; ganesh.acharya@ki.se (G.A.); purusotam.basnet@uit.no (P.B.); 6 Department of Clinical Science, Intervention and Technology (CLINTEC), Karolinska Institutet, 171 77 Stockholm, Sweden; 7 Women’s Health and Perinatology Research Group, Faculty of Health Sciences, UiT The Arctic University of Norway, Universitetsveien 57, 9037 Tromsø, Norway

**Keywords:** resveratrol, chitosan, liposomes, hydrogel, *Chlamydia trachomatis*, antimicrobial, vaginal therapy

## Abstract

*Chlamydia trachomatis* is the most common cause of bacterial sexually transmitted infections and causes serious reproductive tract complications among women. The limitations of existing oral antibiotics and treatment of antimicrobial resistance require alternative treatment options. We are proposing, for the first time, the natural polyphenol resveratrol (RES) in an advanced delivery system comprising liposomes incorporated in chitosan hydrogel, for the localized treatment of *C. trachomatis* infection. Both free RES and RES liposomes-in-hydrogel inhibited the propagation of *C. trachomatis* in a concentration-dependent manner, assessed by the commonly used in vitro model comprising McCoy cells. However, for lower concentrations, the anti-chlamydial effect of RES was enhanced when incorporated into a liposomes-in-hydrogel delivery system, with inhibition of 78% and 94% for 1.5 and 3 µg/mL RES, respectively for RES liposomes-in-hydrogel, compared to 43% and 72%, respectively, for free RES. Furthermore, RES liposomes-in-hydrogel exhibited strong anti-inflammatory activity in vitro, in a concentration-dependent inhibition of nitric oxide production in the LPS-induced macrophages (RAW 264.7). The combination of a natural substance exhibiting multi-targeted pharmacological properties, and a delivery system that provides enhanced activity as well as applicability for vaginal administration, could be a promising option for the localized treatment of *C. trachomatis* infection.

## 1. Introduction

*Chlamydia trachomatis* is a major cause of sexually transmitted infections (STIs) with more than 127 million new infections reported globally each year [1]. It is responsible for serious reproductive tract complications among women, affecting their health and wellbeing. Due to its asymptomatic nature, the reported number of infections is probably underestimated. *C. trachomatis* is a small obligate intracellular bacterial pathogen with a specialized biphasic life cycle. The bacterium exists in two forms; the infectious elementary body and non-infectious reticulate body that conceals its antigenic profile from the immune system by replicating in an intracellular vacuole and then moving between the hosts in the non-replicative form [2]. Current treatment of *C. trachomatis* infections includes orally administered azithromycin (AZT) or doxycycline, however, they may have subsequent side effects; moreover, clinical failure and high recurrence rates do represent an increasing concern. In addition, tetracyclines are contraindicated during pregnancy [3]. Although the proofs that antimicrobial resistance (AMR) is a cause of the current therapy failure remains to be widely discussed, there is clear evidence of in vitro heterotypic resistance to conventional antibiotics at high levels of organism load [4]. The extensive use of a single dose of AZT for uncomplicated chlamydia infections is questionable [3], whereas the efficacy of conventional treatment is threatened by the continuous rise of AMR and might become limited [4,5,6]. Treatment failure or even the lack of treatment can be associated with serious consequences such as ectopic pregnancy and tubal infertility [7]. In the search for novel tools to tackle AMR, natural substances with multi-targeted pharmacological properties have emerged as possible alternatives to antibiotics [8], including for the treatment of *C. trachomatis* infections [9,10].

Resveratrol (RES) is a substance of natural origin that is gaining much attention due to its many potential health benefits. Moreover, it is found to be safe and well tolerated [11]; hence, RES is a promising compound for pharmaceutical applications, including those for vaginal administration. In addition to its known anti-oxidative and anti-inflammatory properties, in recent years, its antimicrobial activities highlighted RES as an interesting alternative to antibiotics [11,12]. A wide range of bacterial, viral and fungal species are known to be susceptible to RES, including *C. trachomatis* [13,14]. The anti-chlamydial effect of RES is not yet completely understood, however, inactivation of the efflux pump [15], inhibition of bacterial type III secretion system (T3SS) [16,17,18] and prevention of cell entry by covalently binding to the elementary body of *C. trachomatis* [17] are all suggested as possible mechanisms for bacterial growth inhibition. Since RES is a poorly water soluble substance and has rather limited bioavailability and stability, a suitable delivery system is required to obtain optimal therapeutic effect [19]. When aiming for localized treatment of vaginal infections, the delivery system must hold the necessary characteristics needed to assure a uniform distribution throughout the vaginal cavity and a sufficient drug concentration at vaginal site for an adequate amount of time. Simultaneously, the system should improve the solubility of RES and protect the active ingredient to assure its stability and activity at vaginal site. We therefore proposed RES liposomes-in-hydrogel as a superior formulation targeting vaginal chlamydia infection.

The proposed delivery system comprises RES liposomes incorporated into chitosan hydrogels, where chitosan hydrogel enables an appropriate distribution and assures a prolonged contact time at the vaginal site, whereas liposomes tackle the solubility and stability issues of RES. The combination of the two delivery systems should enhance the antimicrobial effect of RES and enable a successful localized treatment. We therefore, as the first stage, challenged the novel RES liposomes-in-hydrogel formulation against *C. trachomatis* using the commonly applied cell line for chlamydial infections, namely McCoy cells [14,20], under in vitro conditions. Further, its anti-inflammatory activity was evaluated to support the superiority of the novel formulation.

## 2. Materials and Methods

### 2.1. Materials

Lipoid S 100 (>94% phosphatidylcholine) was provided by Lipoid GmbH, Ludwigshafen, Germany. Chitosan (medium molecular weight hydramer HCMF (350–600 kDa), degree of deacetylation 70–95%) was provided by Chitinor, Tromsø, Norway. McCoy mouse fibroblast (ECACC 90010305), Resveratrol (RES: 3,5,4′-trihydroxy-trans-stilbene, purity ≥99%), acetic acid, glycerol, Dulbecco’s modified eagle medium (DMEM)—high glucose, Hank’s balanced salt solution, cyclohexemide, glutamine, sodium phosphate, sucrose, potassium dihydrogen phosphate, glutamic acid, propylene glycol, RPMI 1640 medium and bovine serum albumin were the products of Sigma-Aldrich, Chemie GmbH, Steinheim, Germany. *Chlamydia trachomatis* Serovar E (ATCC^®^ VR-348B™) was purchased from LGC Standards GmbH, Wesel, Germany. Pathfinder™ Chlamydia Culture Confirmation System was the product of Bio-Rad Laboratories AB, Oslo, Norway. Azithromycin (AZT) was a gift from PLIVA Croatia Ltd., Zagreb, Croatia. Fetal Bovine Serum (FBS) and lipopolysaccharide (LPS; Escherichia coli, 055:B5) was purchased from Sigma Life Science (Sigma—Aldrich Norway AS, Oslo, Norway). Griess reagent: phosphoric acid, sulfanilamide and naphthylethylenediamine dihydrochloride were all products of Sigma-Aldrich Norway AS, Oslo. Murine macrophages RAW264.7 cells were from ATCC, Manassas, VA, USA. Uranyless was the product of Electron Microscopy Sciences, Hatfield, PA, USA. All chemicals were of analytical grade.

### 2.2. Preparation of RES Liposomes

Liposomes were prepared by the film hydration method [19]. Briefly, RES (10 mg) was dissolved in ethanol and Lipoid S 100 (200 mg) was dissolved in methanol. The solutions were mixed and solvents were removed by evaporation (Büchi rotavapor R-124 with vacuum controller B-721, Büchi Vac^®^ V-500, Büchi Labortechnik, Flawil, Switzerland) for 3 h at 50 mm Hg and 50 °C. Distilled water (10 mL) was added to the dry lipid film and shaken to generate liposomes. Liposomal suspension was stored in a refrigerator (4–8 °C) overnight prior to further use. Plain liposomes (not containing RES) were prepared in similar manner.

Extrusion through polycarbonate membranes (Nuclepore Track-Etch Membran, Whatman House, Maidstone, UK) was applied for vesicle size reduction [21]. This was done stepwise through 0.4, 0.2 and 0.1 µm pore size filters, each step repeated 5 times. Extruded liposomes were stored in a refrigerator overnight, prior to characterization and further use.

### 2.3. Characterization of RES Liposomes

Liposomal size distribution was determined by photon correlation spectroscopy (Submicron particle sizer model 370, Nicomp, Santa Barbara, CA, USA). All sample preparations were done in a laminar airflow bench and liposomal size analyses were run in vesicle mode and intensity-weight distribution. Three parallels, with a run time of 10 min, were done for all samples. 

Malvern Zetasizer Nano Z (Malvern, Oxford, UK) was used in the determination of zeta potential as previously described [22]. Samples were diluted in filtrated water (0.2 µm) to appropriate concentrations as previously described. Prior to measurements, the Zetasizer measurement cells (DTS1060) were rinsed with ethanol and filtrated water, respectively. Three parallels were measured at 25 °C for all samples. 

Dialysis was applied to separate free RES from liposomally entrapped RES (Mw cutoff: 12–14,000 Da, Medicell International Ltd., London, UK). Distilled water was used as the dialysis medium in a ratio of 1:250 mL to assure the solubility of RES [19]. After 4 h of dialysis, aliquots of both sample and dialysis medium were diluted in methanol and measured spectrophotometrically (Microtitre plate reader; Spectra Max 190 Microplate, spectrophotometer, Molecular Devices, Sunnyvale, CA, USA) at 306 nm to determine RES entrapment efficiency.

The morphology of RES liposomes was inspected by transmission electron microscopy (TEM). Liposomal formulations were deposited on glow discharged 200 or 400 mesh carbon-coated grids for 5 min. The samples were stained with uranyless for 10–40 s and air-dried for 30 min prior to imaging, using the HT7800 (Hitachi, Tokyo, Japan) at 20–120 kV acceleration voltage equipped with a Morada digital camera.

### 2.4. Preparation of RES Liposomes-In-Hydrogel Formulation

Chitosan hydrogels were prepared as previously described [8]. In brief, medium molecular weight chitosan (350–600 kDa, DD 70–95%) was dispersed in a mixture of 1% (*w*/*w*) acetic acid and 10% (*w*/*w*) glycerol, to a final polymer concentration of 2.5% (*w*/*w*). The hydrogels were left to swell at room temperature (23–25 °C) for 48 h. Liposomes containing RES (free of unentrapped RES) were evenly distributed in the chitosan hydrogel by careful hand stirring. Formulations with a final concentration of 20% (*w*/*w*) liposomal suspension were prepared, corresponding to 166–174 µg/g RES in formulation.

### 2.5. In Vitro RES Release

RES release was determined by a spiral release system (developed at the University of Freiburg, Freiburg, Germany) which is suitable for the release studies of semi-solid formulations such as hydrogels [23]. Formulations and controls (20 g, containing 3.3–3.5 mg of RES) were weighed in the donor part of the system and connected to the receptor part, separated by the cellophane membrane. Receptor medium (400 mL acetate buffer, pH 4.6) was kept at a temperature of 37 °C and continuously flushed through the receptor part of the system (at 100 rpm). Samples (500 µL) were taken after 1, 2, 3, 4, 6 and 8 h and replaced with fresh buffer. RES content in all samples was measured spectrophotometrically at 306 nm.

### 2.6. McCoy Cell Culture and Propagation of Chlamydia trachomatis

McCoy cells were grown in high glucose Dulbecco’s modified Eagles’ medium (DMEM) supplemented with 10% heat inactivated fetal bovine serum (FBS) and 2 mM glutamine in Corning^®^ T75 flasks (Sigma-Aldrich). To avoid the synergistic effect of antibiotics and RES, the cells were grown in the absence of antibiotics for 5 passages before aliquoted in 24-well plates. Cell monolayers were infected with *C. trachomatis* diluted in SPG (250 mM sucrose, 10 mM sodium phosphate, 5 mM potassium dihydrogen phosphate, 5 mM glutamic acid). Plates were centrifuged for 1 h at 2000× *g* and incubated for 1 h at 37 °C and 5% CO_2_. Media was replaced with fresh media supplemented with additional 5% glucose and 0.5 µg/mL cycloheximide and incubated for 48 h at 37 °C and 5% CO_2_. For the cells receiving treatment: RES liposomes, RES liposomes-in-hydrogel, respective controls and positive control (AZT) were prepared in the fresh media in the RES concentrations of 1.5, 3 and 6 µg/mL, respectively. The treatment was added 2 h post-infection and the cells were incubated further at 37 °C and 5% CO_2_. The media was removed and methanol was added to fix the cells at 48 h post-infection. The Pathfinder™ Chlamydia Culture Confirmation System (Bio-Rad Laboratories AB, Oslo, Norway) was used in staining of the inclusions [24]. The plates were inspected under fluorescence microscope (Leica DMI6000B microscope with Leica DFC320 digital camera, Leica Microsystems, Wetzlar, Germany) and 5 pictures (10×) were taken in similar areas of each well to determine the number of inclusions. Susceptibility testing was performed in duplicate and the experiment was performed in quadruple.

### 2.7. Inhibition of NO Production

Anti-inflammatory activity of RES formulation was expressed by measuring its inhibition activity on the nitric oxide (NO) production in LPS-induced macrophage (RAW 264.7 cells), as described earlier [25]. Macrophages (1 × 10^5^ cells/well) were incubated in a 24-wells plate with RPMI 1640 medium with additional 10% fetal bovine serum (FBS) and glutamine at 37 °C and 5% CO_2_. After 24 h, the medium was replaced with fresh media containing 1 µg/mL of LPS to induce inflammation. Media (10 µL) was used as the negative control and media with LPS (10 µL) as the positive control. The test samples of various formulations were homogenized in media to obtain the RES concentrations of 50, 500 and 2500 ng/mL that were added to the wells containing cells with LPS media. Cells were incubated for 24 h at 37 °C and 5% CO_2_ to observe anti-inflammatory effects of the samples. Media (0.3 mL) was collected from each well, mixed with an equal volume of Griess reagent (1% sulfanilamide, 0.1% naphthylethylenediamine dihydrochloride, 2.5% phosphoric acid) and incubated for 30 min [8]. The macrophage challenged with LPS produced NO which was stabilized as nitrite in the media, corresponding to NO produced by the cells. The nitrite in media after the reaction with the Griess reagent was measured spectrophotometrically (Agilent Technologies, Santa Clara, CA, USA) at 540 nm. The relative anti-inflammatory effects of the formulations on the inhibition of NO production was expressed in a percentage, compared to corresponding controls (cells treated with 1 µg/mL LPS).

### 2.8. Statistical Analyses

We used the student’s *t*-test to compare the means. A *p*-value less than 0.05 was considered statistically significant.

## 3. Results

### 3.1. Characteristics of Liposomes-in-Hydrogel Formulation

The liposomes, both plain and RES liposomes, were characterized by measuring the vesicle size, size distribution, zeta potential and RES entrapment efficiency (Table 1). The liposomes were found to be in the desired size range between 100 and 200 nm [8], with average vesicle size of 156 ± 21 nm and 158 ± 22 nm for plain and RES liposomes, respectively. The low polydispersity index (PDI, 0.073 and 0.077 for plain and RES liposomes, respectively), indicated that the majority of liposomes were of similar size. The obtained entrapment efficiency of 85% (from starting amount of RES) was quite high and also corresponded to our own and literature data [8,19,26,27,28]. Both plain and RES liposomes exhibited a close to neutral zeta potential (−0.56 ± 0.86 mV and −6.72 ± 2.47 mV for plain and RES liposomes, respectively), as expected [8,19]. TEM images (Supplementary Figure S1) of RES liposomes displayed a spherical morphology and size corresponding to the size reported in Table 1.

**Figure 1:**
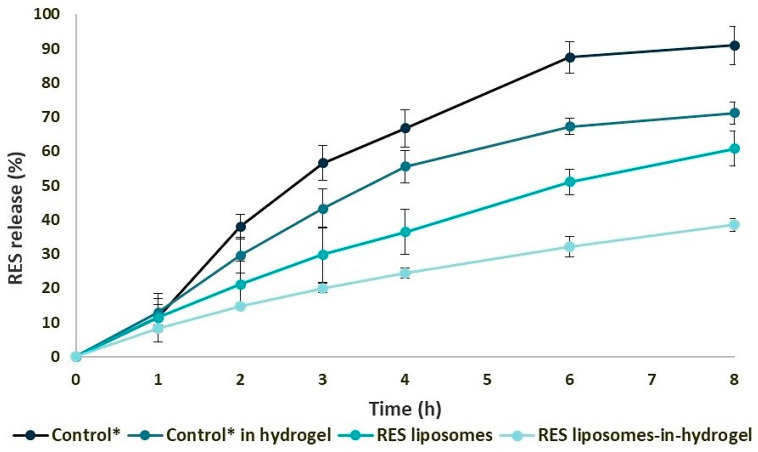
Sustained in vitro resveratrol (RES) release (*n* = 3). RES release from liposomes and liposomes-in-hydrogel compared to the respective controls, assessed by a spiral release system for 8 h. Results are expressed as percentage mean ± SD. * RES in propylene glycol.

Based on optimal texture properties of liposomes-in-hydrogel for vaginal application [8], RES liposomes (20%, *w*/*w*) were incorporated into chitosan hydrogel with a final polymer concentration of 2.5% (*w*/*w*). To assure that RES was indeed available to exhibit antimicrobial action, both formulated as RES liposomes or as RES liposomes-in-hydrogel, the in vitro RES release from RES liposomes and RES liposomes-in-hydrogel formulation was compared to the amount of RES from solution (control; RES in propylene glycol) permeating the membrane. To deal with the very limited solubility of RES in water, for RES in solution we used propylene glycol as a solvent to produce the control solution. We were aware that propylene glycol may induce the osmotic effects resulting in enhanced permeation, however, the option of solvents was limited. The amount of RES permeating the membrane, reflecting the RES released from formulations, was measured for up to 8 h considering the overnight application; for the control (free RES), more than 90% (>3050 µg) of RES permeated the membrane as compared to 38% (1300 µg) RES released from RES liposomes-in-hydrogel. Furthermore, the RES release from RES liposomes and RES in hydrogel was measured. After 8 h, more than 70% (>2400 µg) of RES was released from the RES hydrogel, while 61% (2050 µg) of liposomal RES was released. These results correspond to earlier findings assessed in another experimental setup, namely the Franz diffusion cell system [8]. Both liposomal and hydrogel formulations exhibited a sustained RES release that was further enhanced by the combination of the two delivery systems (*p* < 0.005), providing a prolonged release and probable prolonged effect (Figure 1).

### 3.2. C. trachomatis RES Challenge In Vitro

**Figure 2:**
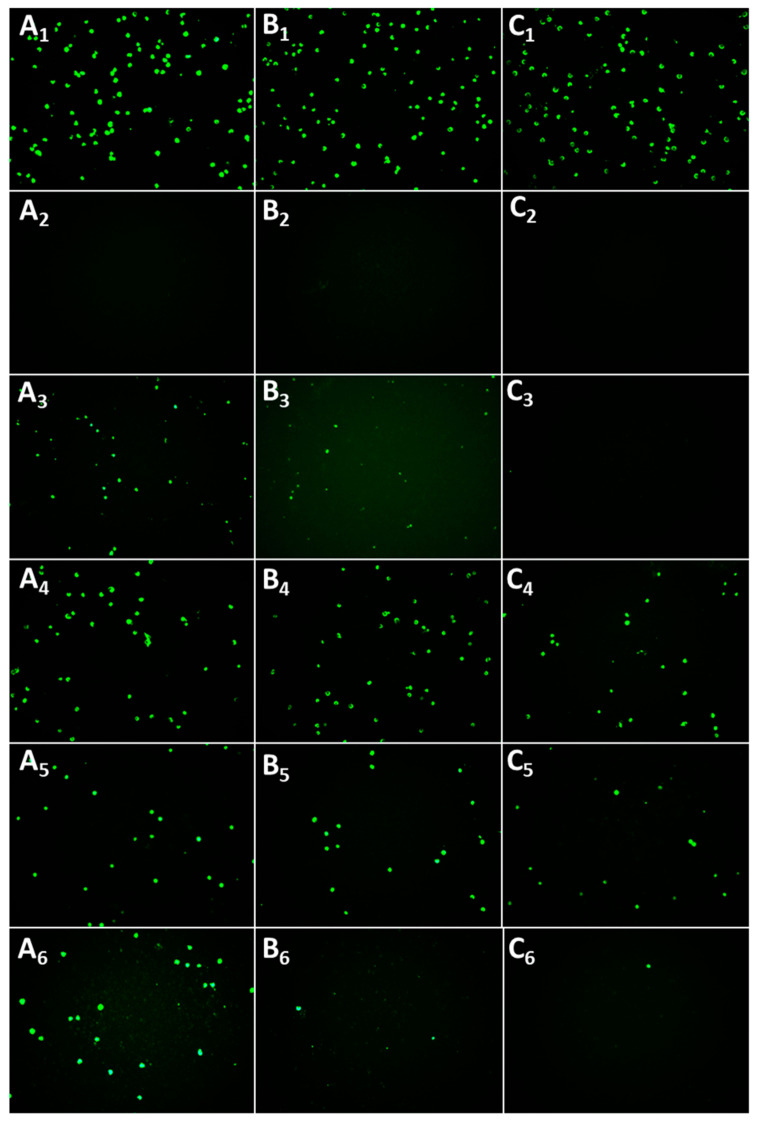
RES formulations reduce the number of *C. trachomatis*-infected cells. Two hours after McCoy cells were infected, the cells were left untreated or added treatment (RES concentrations of 1.5, 3 or 6 µg/mL for (**A**–**C**), respectively). The effect on bacterial survival was evaluated by staining of inclusions. The figure shows representative pictures of the antimicrobial effect of azithromycin (AZT) (2), free RES (control; RES in propylene glycol) (3), RES in hydrogel (4), RES liposomes (5) and RES liposomes-in-hydrogel (6), compared to the negative control (1) where no treatment was added. All pictures were taken with same magnification (10×).

**Figure 3:**
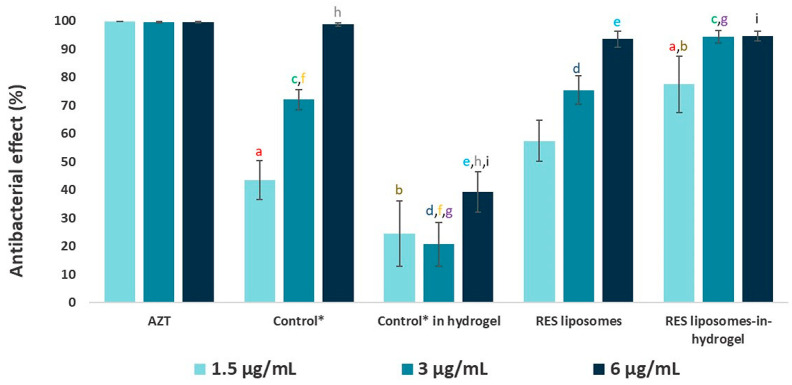
Effect of RES formulations on *C. trachomatis* in vitro (*n* = 4). The values are based on images presented in Figure 2. Number of inclusions for the treated and the non-treated cells were compared. * RES in propylene glycol, ** a**,**b**,**c ***p* < 0.05, **d**,**e**,**f ***p* < 0.005, **g**,**h**,**i** *p* < 0.001.

*C. trachomatis* has been reported to be susceptible to RES [13,14]. However, the solubility and stability of polyphenol RES are limited and a delivery system is needed when considering the potential use of RES as a localized antimicrobial treatment. The ability of RES in different formulations to interfere with *C. trachomatis* viability was therefore tested. The commonly prescribed oral antibiotic AZT was used as a positive control; as expected AZT completely reduced the bacterial growth. A clear reduction in the number of *C. trachomatis*-infected cells was observed after treatment with RES (Figure 2 and Figure 3).

RES liposomes-in-hydrogel expressed superior anti-chlamydial activity in the lower concentrations with inhibition of 78%, 94% and 95%, for 1.5, 3 and 6 µg/mL RES, respectively, compared to 43%, 72% and 99%, respectively, for the free RES (control). Moreover, RES liposomes exhibited a dose-dependent anti-chlamydia effect with inhibition of 57%, 75% and 94%, for 1.5, 3 and 6 µg/mL RES, respectively. However, the RES in hydrogel showed a reduced effect compared to free RES (Figure 3). As expected, plain liposomes, plain chitosan hydrogel and plain liposomes-in-hydrogel did not show any significant inhibition of *C. trachomatis* (2%, 7% and 6% inhibition at 6 µg/mL, respectively), indicating that the delivery systems alone do not exhibit anti-chlamydia effect (data not included in figures). However, when comparing the antibacterial effect of free RES (control) and RES liposomes-in-hydrogel, the activity of RES at lower concentrations is clearly enhanced (*p* < 0.05, for both 1.5 and 3 µg/mL) when RES is formulated in the liposomes-in-hydrogel delivery system. None of the tested formulations showed any visible effect on cell count and viability, in agreement with earlier studies that indicated that the RES liposomes-in-hydrogel formulation is non-toxic at lipid concentrations up to 50 µg/mL [8]. 

### 3.3. Anti-Inflammatory Effect of RES Liposomes-In-Hydrogel

We have previously reported on the anti-inflammatory properties of RES and RES liposomes where the NO production in LPS-challenged macrophage was strongly inhibited, and RES liposomes formulation was found to be more potent than free RES [19]. However, the anti-inflammatory effects of RES liposomes-in-hydrogels has not yet been reported. We therefore studied the effect of free RES (control), RES liposomes-in-hydrogel, RES in hydrogel and RES liposomes on the inhibition of NO production. The NO production in the LPS challenged cells was inhibited in a concentration-dependent manner by all formulations. The effect of RES when formulated in hydrogel was clearly enhanced compared to the control, hence, the effect of plain hydrogel and plain liposomes-in-hydrogel was included in testing. The result showed a pronounced inhibition of NO production of the delivery system even without the active ingredient, however, the hydrogel alone expressed limited or no reduction at the lower concentrations. The NO production inhibition of RES liposomes-in-hydrogel was superior to both RES in hydrogel and plain liposomes-in-hydrogel for the higher concentrations tested (Table 2, *p* < 0.05).

## 4. Discussion

In the era of AMR, the search for novel antimicrobials has intensified. Natural origin substances with known antimicrobial activities offer the possibility to act as alternatives to antibiotics or a means to increase the efficacy of conventional antibiotics [29]. Due to its anti-oxidative, anti-inflammatory and antimicrobial properties, the natural polyphenol RES could be the ideal active molecule. The antimicrobial effects of RES have been recently reported [11,30] and over the last few years, the number of publications on RES in various delivery systems increased significantly [31,32,33,34,35]. Due to its poor solubility and limited stability, there is a need for a delivery system that can protect RES from environmental and chemical changes, as well as enable its depot at the site of action to fully utilize its therapeutic properties [36]. In addition, by efficiently treating the infections locally while avoiding the systemic exposure to antimicrobials, we could reduce the side effects and improve its safety, as well as limit the chance for development of AMR. However, the vaginal site comprises intrinsic challenges [8] that need to be overcome by development of smart formulations which enhance the efficacy while improving the stability of RES. The localized treatment of vaginal infections requires a delivery system with suitable properties to assure a uniform distribution over vaginal tissue, a sufficient contact time and an adequate drug concentration at targeted site [37]. Chitosan hydrogels have a low pH (4–5), are biodegradable and exhibit excellent mucoadhesive properties; these are qualities that are considered desirable for vaginal drug delivery [38]. Various nanocarriers have the ability to increase the potency of active substances and constitute an important part in the treatment of infectious diseases [39]. Liposomes are known to improve RES solubility and to provide its sustained delivery [8,19,26,32]. Furthermore, liposomes are shown to improve its chemical stability, enabling therapeutic benefits [40,41,42]. Incorporation into liposomes allows RES to better merge with bacterial cells, resulting in an improved antimicrobial effect, even at lower doses [40]. Liposomes were also able to protect incorporated antibiotics, such as vancomycin, from degradation and increase its potency against bacteria [43]. Recently, the local therapy by liposomes containing AZT, which is commonly used as an oral single-dose in chlamydia treatment, has shown efficacy against cervicovaginal bacterial infections [44]. Moreover, incorporation of liposomes into hydrogels has shown an ability to assure hydrogel stability [8], and hydrogels in liposomes-in-hydrogels act on improving the stability of incorporated liposomes by preserving their original vesicle size [45]. 

### 4.1. RES Liposomes-In-Hydrogel Formulation

To assure successful localized treatment of vaginal infections such as chlamydia, the delivery system should provide an adequate drug concentration at the site of infection for a sufficient amount of time. Several studies support the hypothesis that the liposomal formulation of RES improves its therapeutic benefits by improving its solubility and chemical stability [41,42,46]. Moreover, the storage stability of liposomes can be improved by polyphenol incorporation, due to their inherent anti-oxidative properties [41]. An appropriate liposomal vesicle size for local administration is necessary to enable a depot effect and avoid a systemic effect. The reduction of vesicle size can lead to a loss of originally entrapped material, in our case the RES load, however, the smaller vesicle size did not compromise the RES entrapment efficiency that was found to be sufficiently high, with an average of 85% RES in liposomes (Table 1). Additionally, the very low polydispersity index (PDI) denotes a narrow liposomal size distribution. The incorporation of liposomes into chitosan hydrogel has shown improvement of the gel’s texture properties [8] as well as providing an appropriate viscosity for vaginal application [47]. The cohesiveness and adhesiveness of hydrogels were reduced in the presence of vaginal fluids, however, liposomes were shown to stabilize the chitosan network [48]. Considering the incorporation of neutral liposomes into chitosan hydrogel, that comprises positively charged chains, potential disturbance of the liposomal membrane and rapid release of the entrapped substance is avoided [49]. Accordingly, a consistent and sustained RES release from liposomes-in-hydrogel formulation was confirmed (Figure 1), in agreement with earlier findings utilizing the Franz diffusion cell system [8]. Although both liposomal and hydrogel formulations exhibited a sustained RES release, the release was further sustained by the combination of the two delivery systems. This indicates a synergy effect on the release, and contributed to the fact that the RES has to be released from the liposomes first, followed by release from hydrogel in the case of the RES liposomes-in-hydrogel formulation. Considering the potential biological activities of RES, the concentration of released RES from the liposomes-in-hydrogel system is above the effective concentration required to induce the desired effect in the *C. trachomatis* challenge in vitro. The in vitro release study presented in Figure 1 revealed that the amount of RES released from the liposomes-in-hydrogel formulation (after 8 h) was more than 1300 µg (~38% of the starting amount). For the formulation volume applied in anti-chlamydial challenge (starting RES concentration of 6 µg/mL), the RES amount released after 8 h would correspond to 2.3 µg (2.3 µg/mL). Our results in Figure 3 indicate a potent anti-chlamydial activity at RES concentration as low as 1.5 µg/mL. We therefore expect that the amount of RES released from the formulation is above the required effective concentration. The use of hydrogels as vaginal formulations, due to the sustained release of active substances has a consequent therapeutic effect which is favorable [50]. Additionally, we have earlier confirmed in an ex vivo RES penetration study through sheep vaginal tissue a very limited RES penetration from liposomes (4.5–5%), while the majority of RES was found within or retained on the tissue [8], confirming the lack of its systemic effect. This would enable safe administration of RES formulations to pregnant patients as well. 

### 4.2. Anti-Chlamydial Activity

The ability of RES to inhibit propagation of *C. trachomatis* in McCoy cells was recently reported [14]. RES assured a dose-dependent reduction in the number of infected cells. The liposomes-in-hydrogel system is expected to enhance the RES activity, especially considering that RES has to be protected from environmental and chemical changes to exert full therapeutic potential [34]. Accordingly, RES liposomes-in-hydrogel exhibited potent anti-chlamydial activity (Figure 2 and Figure 3) and was found superior to free RES (control) in lower concentrations (*p* < 0.05). Additionally, liposomal RES expressed a high inhibition of *C. trachomatis*, confirming that the effect of RES is not reduced by liposomal entrapment. Literature supports the finding that liposomal entrapment of RES improves its ability to merge with bacterial cells, leading to an enhanced antimicrobial effect [40]. However, RES in hydrogel did not succeed to inhibit the propagation of *C. trachomatis* (Figure 3). This is a strong indication that the ability of liposomes to assure RES stability and protect the incorporated RES is of importance for its therapeutic effect. Considering the conditions at vaginal site, the mucoadhesive properties assured by chitosan hydrogel are necessary [48], and studies show that the use of hydrogels is essential when aiming for improved therapy by the vaginal route [51]. Recent findings confirmed that chitosan hydrogel has intrinsic antibacterial activity [48], however, *C. trachomatis* was not inhibited by chitosan hydrogel on its own. It is therefore evident that for full utilization of RES as a potential anti-chlamydial treatment, both liposomes as a primary delivery system and chitosan hydrogel as a secondary vehicle are required.

We are highly encouraged by the in vitro findings, however, the results need to be explored in vivo to determine clinical relevance. In parallel, the exact mechanism of bacterial growth inhibition by RES should be fully mapped to assist in the optimization of the vehicle to be used to deliver RES into the infected cells [11,14]. The mechanism of antibacterial action of RES is not completely understood, however, the inhibition of the ATP synthase, DNA fragmentation and membrane damage might be involved [11,12,52]. Regarding the inhibition of *C. trachomatis*, the inclusion membrane presents an additional barrier that has to be crossed to reach the intracellular bacteria. RES is generally found to be less active against gram-negative bacteria compared to gram-positive, possibly due to the efflux pump systems in gram-negative species [11,53]. Hence, the inactivation of the efflux pump can increase the antibacterial activity of RES [15]. Moreover, *C. trachomatis* possesses a bacterial type III secretion system (T3SS), which might facilitate the entry of bacteria into the host cell, thus presenting a target for bacterial growth inhibition [16,17,18]. Several monomers, dimers and oligomers of RES have shown to inhibit the T3SS in various bacteria [54], including *C. trachomatis*. Although the *C. trachomatis* inclusion membrane presents an additional barrier, it also has low permeability for larger compounds, and Zetterström and colleagues hypothesized that the RES tetramer (-)-hopeaphenol covalently binds to the elementary body of *C. trachomatis* and thereby prevents cell entry and intracellular replication [17]. In the present work, the treatment was added post-infection, which implies that RES affects the intracellular stage of the *C. trachomatis* life-cycle. This indicates that the incorporation into liposomes allows RES to interfere with bacterial cells, achieving an improved antimicrobial effect at lower doses. It is assumed that liposome membranes fuse with the cell membranes, improving the delivery of RES to the cells [40]. It is worth noting that lipids in liposomes, chitosan and RES are all of natural origin and they are confirmed to be non-toxic [8]. Liposomes are also considered to be favorable nanocarriers when aiming for localized treatment, as they do not interfere with the vaginal microbiota [55].

RES liposomes-in-hydrogel offers an additional advantage which is currently one of our interests. Namely, vaginal infections are often associated with the formation of biofilms which can decrease the susceptibility to antimicrobial agents and increase the hazard of AMR. Nanoparticles have shown an ability to penetrate biofilms and have great potential in the treatment of challenging infections [56]. Moreover, since chitosan hydrogel efficiently disrupts vaginal biofilms [57], while RES inhibits the bacterial biofilm formation [58], the proposed RES liposomes-in-hydrogel might be targeted and utilized for the treatment of other types of vaginal infections.

### 4.3. Anti-Inflammatory Activity

Vaginal inflammation is very common and often associated with vaginal infection, including by bacteria such as *C. trachomatis*. Cervical cells infected with *C. trachomatis* activates the release of pro-inflammatory cytokines [59]. Some free radicals, such as nitric oxide (NO^•^), are responsible for mediating the inflammation; we therefore tested the ability of RES formulations to inhibit NO production. We reported earlier that liposomal RES exhibited a stronger inhibition of both NO and TNF-α production in LPS-induced macrophages compared to the free RES [15]. The findings in the current study show that the effect of RES upon incorporation into formulation (liposomes-in-hydrogel) was clearly enhanced (*p* < 0.05) compared to free RES (Table 2). Interestingly, the effect of RES when in hydrogel was also noticeably enhanced. Consequently, we evaluated the ability of plain liposomes-in-hydrogel to inhibit NO production; a relatively high anti-inflammatory effect was measured for the delivery system without RES. However, the hydrogel on its own expressed anti-inflammatory activity only at the highest concentration. The superior inhibition determined for RES liposomes-in-hydrogel, as compared to both RES in hydrogel and plain liposomes-in-hydrogel, suggests a synergy of the liposomes-in-hydrogel formulation and the incorporated active ingredient. The ability of the novel formulation to provide an anti-inflammatory effect would be highly beneficial, considering the success of treatment.

## 5. Conclusions

Liposomes-in-hydrogel formulation containing the natural substance RES as the active ingredient with high RES load was prepared, assuring sustained RES release. The incorporation of RES into the liposomes-in-hydrogel delivery system clearly enhanced the anti-chlamydial effect of RES at lower concentrations, indicating the need for a delivery system to assure successful therapy. Moreover, the delivery system enhanced the anti-inflammatory activity of RES. Finally, the use of alternative antimicrobials, such as RES, can preserve available antibiotics as efficient drugs in the future.

## Figures and Tables

**Table 1:** Characteristics of plain and RES liposomes. Results are expressed as mean ± SD (*n* = 3).

Formulation	Vesicle Size (nm)	PDI *	Zeta Potential (mV)	Entrapment Efficiency (%)
Plain liposomes	156 ± 21	0.073	−0.56 ± 0.86	-
RES liposomes	158 ± 22	0.077	−6.72 ± 2.47	85 ± 2

* Polydispersity index.

**Table 2:** Effect of RES formulations on the NO production expressed as the percentage of reduction (*n* = 3). Concentration of formulations is expressed as RES concentrations (50, 500 and 2500 ng/mL).

RES Formulation	50 ng/mL	500 ng/mL	2500 ng/mL
Control *	3 ± 3	11 ± 2	24 ± 1
RES liposomes-in-hydrogel	15 ± 2	37 ± 5 ^a^	58 ± 4 ^b^
Control * in hydrogel	10 ± 3	18 ± 3 ^a^	51 ± 1 ^b^
RES liposomes	9 ± 2	12 ± 2	16 ± 6
Plain liposomes-in-hydrogel **	17 ± 5	21 ± 1	46 ± 3
Plain hydrogel **	0 ± 1	7 ± 4	54 ± 3

* RES in propylene glycol, ** adjusted to concentration in formulations, ^a^ *p* < 0.005, ^b^ *p* < 0.05.

## Supplementary Materials

The following are available online at https://www.mdpi.com/1999-4923/12/12/1203/s1, Figure S1: TEM image of RES liposomes.
