# Supplementary material for: Liposomes-In-Hydrogel Delivery System Enhances the Potential of Resveratrol in Combating Vaginal Chlamydia Infection
Source: Pharmaceutics. 2020 Dec 11;12(12):1203. doi: 10.3390/pharmaceutics12121203 (PMC7764002; doi:10.3390/pharmaceutics12121203)
Supplement: Supplementary file 1 [file pharmaceutics-12-01203-s001.pdf]

Supplementary Materials: Liposomes-in-Hydrogel Delivery System Enhances the Potential of Resveratrol in Combating Vaginal Chlamydia Infection

May Wenche Jøraholmen, Mona Johannessen, Kirsten Gravningen, Mirja Puolakkainen, Ganesh Acharya, Purusotam Basnet, Nataša Škalko-Basnet

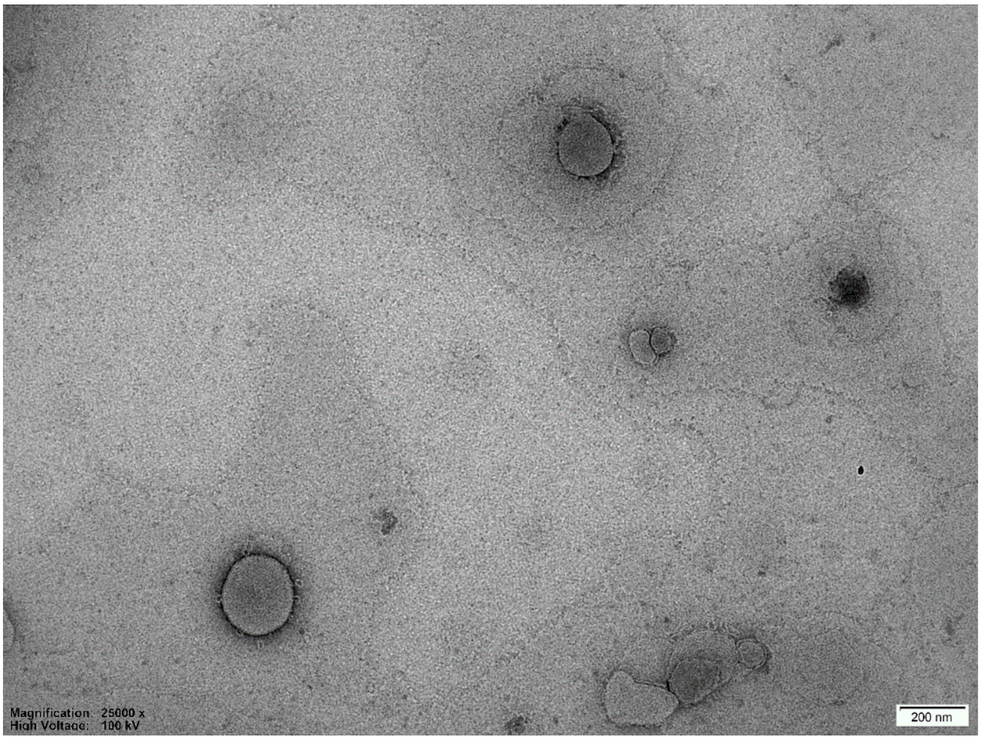

Figure S1: TEM image of RES liposomes.
